# Why Did He Say This?

**Published:** 1898-09

**Authors:** 


### EDITORIAL.

### Why Did He Say This?

In a recent number of the *American Journal of Dental Science* Dr. Ottolengui, Editor of the Items of Interest, says: "The professional quack is a miserable sort of fellow. He takes a notion that he would like to be a dentist. (1)* He has little or no education, but in these days that is no obstacle, providing he has a couple hundred of dollars. (2) Any dental college in the United States will accept him as a student (vide Proceedings National Association of Dental Faculties). (3) He worries along through college to the examination period, and this he passes because he wears long white cuffs, and knows how to write a fine legible hand with a well-pointed pencil. So he gets through and is awarded a diploma. He does not send that pair of cuffs to the laundry, but uses them at the State Board examination, which he passes with the same facility, and with the same lack of knowledge, as when going through college. The College and State Board have done all they could for him; they have launched him. But thrown upon his own resources he finds that he has none. * * * * His leaning toward mechanics made him imagine himself fitted for dentistry, and when he applied for admission into college the professors did not undeceive him."

In reading these statements by Dr. Ottolengui the question naturally arises, "Why did he say these things?" Was it because he *knew* that they were true? Did he write them because of his knowledge of the facts or because he supposed they were true, or did he write from the statements of some other, no better posted than himself?

Statement No. 1, made above, lacks very much in correctness. As far as may be inferred from the statements made, all dental colleges are in the same category, as no exception is made. In reference to the large majority of the colleges having membership in the National Association of Dental Faculties, this statement is not true, and no man has a right to make this statement in regard to any college in this Association unless he has absolute knowledge, and then he ought to be specific.

The statement *No.* 2 is a flagrant violation of the truth, as every college man in the country knows, and as every editor of a dental journal ought to know, and almost every dentist worthy of the name ought to know. These sweeping statements are aimed at the colleges that have membership in the National Association of Dental Faculties.

Statement *No.* 3, "He worries along through college to the examination period, and this he passes," according to Dr. Ottolengui, not because he has studied, or has learned anything, but because he "wears long white cuffs and knows how to write a fine legible hand with a well-pointed pencil." Here is a requirement that is new to almost everybody. It is a sad commentary upon the college students, and college teachers in the country, that they will graduate students because they wear "long white cuffs and knows how to write a fine legible hand with a well-pointed pencil."

"So he goes through and is awarded a diploma," not because he has been taught anything, but because he wears "long white cuffs." He is greatly interested in his cuffs, takes great care of them, as they are to serve him in another emergency. He goes before the State Dental Examining Board, and these same "long white" cuffs enables him to pass the ordeal with the same facility, and with the same lack of knowledge as when going through college.

What a blind, ignorant set of teachers our colleges must have, and the members of our examining Boards as well, that they can be so egregiously imposed upon by the "long white cuffs!"

"The college and State Board have done all they could for him; they have launched him. He is thrown upon his own resources and finds he has none." And yet he is thrown upon them. What a remarkable statement! A man being thrown upon something that does not exist.

"He imagines himself fitted for dentistry, and when he applies for admission into the colleges the professor does not undeceive him." A very wrong thing for professors to do. It is clear that those whom Dr. O—— knows are very naughty professors.

In looking over these statements, we are greatly surprised that Dr. Ottolengui should have made them. We are nearly as much surprised to see that the *American Journal of Dental Science* has published such statements, and a good deal surprised that the *British Journal of Dental Science* also would publish them. We can only account for the first publication by the supposition that the editor did not examine his copy or read the proof.

Why any one occupying the position of Dr. Ottolengui should write such tirade and untruth against the educational system and institutions of his own profession, of his own country, is astounding.

If there were any suggestions for improvement, or any indica tions of sympathy for the educational efforts in our profession, some of these statements might be overlooked. But such is not the case in the article under consideration. It is an arraignment of the teachers of our colleges and the members of our examining boards that is unwarranted by the facts.

